# Antibiotic Resistance

**DOI:** 10.1155/S106474499700063X

**Published:** 1997

**Authors:** Sebastian Faro


Infectious Diseases in Obstetrics and Gynecology 5:359 (1997)
© 1998 Wiley-Liss, Inc.

### Antibiotic Resistance

Antibiotic resistance is an ongoing evolutionary process that has been the concern of infectious disease specialists since antibiotic use gained popularity. Antibiotic resistance is not a concern of the public as reflected by their desire for antibiotics whenever illness occurs. Patients can and do pressure their physicians for antibiotics; 12 million antibiotic prescriptions were filled for upper respiratory tract infections and bronchitis in 1992 (Gonzales R, et al. JAMA 278:901, 1997). Many physicians disregard the emergence of resistance as a phenomenon that occurs, but is not found, in their community or hospital and therefore is not a real concern.

Antibiotic resistance is extremely costly. There is an increase in morbidity, prolonged hospitalization, the ability for infections to be disseminated to other individuals, and the loss of human life. There is no doubt that the evolutionary process is continuous in hospitals and communities. The umbrella of community includes both cities and rural farming areas. Farmers use tremendous quantities of antibiotics in crop production and raising livestock. This usage exerts significant selective pressure on bacteria in the environment, which eventually make their way to humans.

The response to this problem has not been to develop monitoring systems, but to introduce new broad-spectrum antibiotics. Following the latter is folly since bacteria have demonstrated, again and again, their ability to overcome new agents by mutation. In addition, gram-negative bacteria have the ability to transfer resistance to other bacteria, even between different genera. This was revealed when gram-negative bacteria adapted to beta-lactamases. Forestalling the evolutionary process can be achieved by reducing the selective pressure by limiting exposure to antibiotics.

William and Heyman (Science 279:1153–1154, 1988) suggested that progress in the development of antibiotic resistance can be accomplished by the following: appropriate human use of antibiotics, decrease or eliminate use of antibiotics for nonhuman indications, and containment of resistant strains in medical facilities. Perhaps an opportunity is at hand to develop a systemwide monitoring program for the infectious disease specialists in obstetrics and gynecology. This appears to be an ideal opportunity for the two societies, IDSOG and IIDSOG, to develop a program for monitoring antibiotic usage as well as educating physicians and patients in their appropriate use.

*Sebastian Faro, MD, PhD*
*Editor-in-chief*
